# Developing the Iranian health insurance benefit optimization model – the IR-HIBOM: a multicriteria decision analysis with decision rules for designing basic health insurance benefit packages

**DOI:** 10.1017/S0266462325100263

**Published:** 2025-07-21

**Authors:** Ali Darvishi, Ali Akbari Sari, Mehdi Yaseri, Mohammadreza Mobinizadeh, Rajabali Daroudi

**Affiliations:** 1 Department of Health Policy and Management, School of Public Health and Safety, Shahid Beheshti University of Medical Sciences, Tehran, Iran; 2 National Center for Health Insurance Research, Tehran, Iran; 3 Department of Health Management, Policy and Economics, School of Public Health, Tehran University of Medical Sciences, Tehran, Iran; 4 Department of Epidemiology and Biostatistics, School of Public Health, Tehran University of Medical Sciences, Tehran, Iran; 5 National Institute for Health Research, Tehran University of Medical Sciences (TUMS), Tehran, Iran

**Keywords:** technology assessment, biomedical, priority setting, insurance benefits package, allocation of public health resources, multicriteria decision analysis, preference assessments

## Abstract

**Objectives:**

Prioritization of health technologies for insurance coverage is usually based on explicit and implicit criteria. This study presents the development of the multi-criteria decision analysis (MCDA) model, the Iranian Health Insurance Benefit Optimization Model (IR-HIBOM), to inform the design of basic health insurance benefit packages.

**Methods:**

An initial set of twenty-nine potential allocation criteria was identified through a review of available evidence and other relevant literature. Review of this set by three specialized panels yielded a final set of thirteen criteria. A cross-sectional survey using the best–worst scaling method was then fielded to 163 health system experts to evaluate their preferences regarding the relative importance of the allocation criteria. The mixed logit method was employed to determine the weight of the relative importance of each criterion. Subsequently, a multilevel criteria scoring framework was defined based on a review of similar models and input from a panel of five expert members of the study team. Finally, model’s appraisal was conducted.

**Results:**

Thirteen criteria, including relative safety, efficacy, disease severity, access to alternative health technologies, budget impacts, cost-effectiveness, quality of evidence, population size, age, job absenteeism, economic status, daily care needs, and ease-of-use/acceptance were selected. Cost-effectiveness and ease-of-use criteria had the highest and lowest relative importance weights, with 30.5 percent and 1 percent, respectively. Furthermore, scores were determined for the several levels of each criterion, and decision rules were defined for the cost-effectiveness and budget impact criteria. The final model’s appraisal, based on weighted scores of thirteen selected technologies, indicated that it was valid and applicable.

**Conclusions:**

The IR-HIBOM demonstrated its potential utility in the health resource allocation.

## Introduction

A critical and challenging issue confronting health technologies decision making lies in the imperative to be more accountable for healthcare spending, particularly within publicly funded health systems. The rapid escalation of health costs necessitates making judicious decisions regarding the coverage of new health technologies and reconsidering currently covered technologies based on updated evidence. Health technology assessment (HTA) has evolved in response to this need (1).

An advantage of HTA and related economic evaluations is their use of incremental cost-effectiveness ratios (ICERs) for comparing different health technologies and to establish ICER thresholds for acceptance of a health technology into health benefits packages. However, as the ICER considers only limited criteria, it may neglect other important ones that are relevant for setting resource allocation priorities among health technologies. This limitation sometimes leads to lowering the relative priority of health technologies deemed more valuable by some stakeholders (2).

Empirical evidence indicates that, when setting priorities for health technologies, policymakers deem as important such criteria as efficiency, equity (e.g., prioritizing severely ill or impoverished individuals), equality, financial protection, and political considerations (3–5). However, policymakers face challenges in simultaneously considering such diverse criteria for decision making due to the uneven availability of supporting evidence, variations in the importance of criteria, potential conflicts among criteria, and difficulties in handling different types of information, leading to the risk of cognitive overload (6). Multicriteria decision analysis (MCDA) offers an approach to overcoming these limitations (6). MCDA allows for the identification of a comprehensive set of criteria, evaluating health technologies based on these criteria in a performance matrix, and then qualitatively or quantitatively assessing the matrix to rank the desired health technologies (6). In the context of prioritizing health technologies, MCDA is an objective, transparent, evidence-based method that aids decision making by considering and evaluating multiple objectives, aspects, and criteria (7).

In Iran’s largely integrated and centralized healthcare system, the Ministry of Health and Medical Education (MoHME) is responsible for policymaking, prioritization, planning, and monitoring activities across the public and private sectors (8). The HTA function in Iran was formalized in 2007 with the establishment of a secretariat under the MoHME. Its mission is to systematically evaluate health technologies to support evidence-based decision making, focusing on aspects such as safety, effectiveness, cost-effectiveness, and broader social, ethical, and legal implications. HTA projects are conducted through partnerships with universities and research centers, supported by a scientific committee that prioritizes topics and oversees evaluations. Additionally, capacity building has been a significant focus, with workshops and a master’s degree program in HTA offered at leading medical universities across the country (9).

The health system’s financing and service provision in Iran follow a hybrid structure, featuring an integrated healthcare network predominantly funded by the government and managed through medical sciences universities under the MoHME’s supervision. Social health insurance is a key element of Iran’s healthcare system, providing health services to about 90 percent of the population (10). Consequently, the compilation and revision of insurance benefits packages and priorities for inclusion of various health technologies in health insurance benefits packages are conducted in tandem. As such, it is imperative to employ practical, transparent, and evidence-based tools.

In developing an MCDA-based methodology, the Iranian Health Insurance Benefit Optimization Model (IR-HIBOM), this study demonstrates a practical tool for evaluating and prioritizing health technologies. These efforts aim to optimize the allocation of public resources for compiling and revising benefit packages and supporting responsible policymaking and decision making, accordingly.

## Methods

The sequential phases undertaken to develop and demonstrate the potential utility of the IR-HIBOM were as follows.

### Extraction and selection of allocation criteria

In the initial phase, the identification and selection of allocation criteria relevant to health technologies prioritization for resource allocation were performed. Various methods are typically employed to identify criteria and other characteristics in preference assessment studies, including one or more of reviewing scientific literature, group discussions, interviews, and expert panels. In this study, a set of criteria from various relevant published studies and other documentation were extracted and summarized through a literature search and evidence review. Notably, given recent published comprehensive reviews of different allocation criteria (11;12), a new systematic review was not conducted in this study. However, an additional set of criteria representing social aspect was extracted based on a survey of Iranian adults conducted by members of the study team (13).

Initially, twenty-nine independent criteria were identified. A single expert panel consisting of five study team members with backgrounds in health economics, health policy, health management, HTA, and epidemiology was established for the review and selection of criteria. Given the large number of candidate criteria, the panel convened across three sessions. The criteria were first categorized into clinical, economic, and social dimensions. During the expert panel sessions, each dimension was thoroughly discussed, and the related criteria were refined and finalized with precise definitions. The finalized set of criteria encompassed conventional criteria from similar studies and novel criteria introducing social aspects not previously included in similar models. In each expert panel session, a subset of criteria was reviewed and discussed. Some criteria were eliminated due to conceptual overlap or low perceived relevance, ensuring that each criterion covered a distinct dimension, while others were retained for further consideration. The consensus process was implemented through a structured prioritization approach: after open discussion, each panel member independently ranked the criteria based on their own preferences and judgment. These individual rankings were then aggregated to produce a collective prioritization. The final set of thirteen criteria was determined based on this multiround, consensus-driven process.

### Preference assessment and ranking of allocation criteria

After the criteria were finalized, the preferences of health system experts in Iran regarding their relative importance for financing health technologies were assessed via an electronic questionnaire. These experts included about 350 qualified individuals, comprising faculty members in healthcare management, health economics, HTA, pharmacoeconomics, physicians, and other selected managers and researchers in the health system. An electronic questionnaire link, accompanied by additional explanations, was distributed via email or social media. To ensure the relevance and credibility of the preference data, expert participants were purposefully selected rather than recruited through open invitations. A complete list of faculty members in these fields was compiled using the national scientometrics database, and email invitations containing the questionnaire link and study details were sent to all of them. In addition, the questionnaire was shared individually via email or secure messaging with a selected group of physicians, pharmacists, and others who held managerial experience within the health system. No open or public invitations were issued through social media platforms; participation was limited to those specifically targeted based on their expertise and institutional roles.

For university faculty members, the questionnaire link, along with detailed explanations and the study objectives, was sent via email. For those faculty members who did not respond to the email, the questionnaire was later shared through social media platforms, specifically WhatsApp and Telegram, to improve the response rate. For others among the qualified individuals, the questionnaire was distributed directly via WhatsApp and Telegram. These platforms were chosen due to their widespread use and accessibility among the intended participants. Ultimately, 171 individuals (48.9 percent) responded to the questionnaire, among whose responses, 163 responses (46.6 percent) were considered valid after eliminating incomplete submissions. All respondents completed the electronic questionnaire independently and remotely. No interviewer facilitation or real-time administration was involved. This self-administered online format allowed respondents to complete the questionnaire at their convenience while maintaining standardization and consistency across responses.

The study determined a minimum necessary time required for participants to thoroughly understand and respond accurately to the questions. As such, eight questionnaires that were completed in a shorter duration than that minimum were deemed invalid and excluded from the final analysis to maintain the integrity of the study results.

#### Best–worst scaling method (BWS)

The BWS method was employed to assess the preferences of the 163 health system experts regarding criteria for prioritizing the financing of health technologies. Utilizing the BWS, experts were asked to identify the best and worst criteria under various choice tasks, engaging in pairwise comparisons between each of these two indicators (best and worst) and other criteria. Subsequently, a maximum–minimum problem was formulated and solved to ascertain the weight of different criteria (14). In this context, the best criterion refers to the one deemed more important than other criteria in any one choice task in the prioritization of technologies for health budget allocation, while the worst criterion is considered the least important compared to other criteria in any one choice task.

Three main types of BWS methods vary in the complexity of the cases or options considered. The choice of method depends on the nature of the study. In this study, the BWS object case method was employed, where the researcher seeks relative values associated with each criterion, without decomposing criteria into their more detailed levels and characteristics (15).

#### Designing scenarios

To match the number of finalized criteria, the questionnaire consisted of thirteen choice tasks, each of which offered a comparison among a subset of four of the thirteen criteria, with each criterion appearing in four choice tasks. The question text remained consistent across all choice tasks. In each question, respondents were required to identify the most important (i.e., “best”) and least important (i.e., “worst”) criteria.

The optimal and balanced selection of choice tasks was achieved using R version 4.2.0 for Windows software.

Online supplementary file 1 presents the full questionnaire and choice tasks related to this section.

#### Data analysis

The collected data underwent analysis using the mixed logit method. The mixed logit model accounts for preference heterogeneity, assuming that parameters are randomly distributed across the population. The β or odds ratio (OR) coefficient signifies the relative preference or importance of a factor (criterion) compared to a reference factor (criterion). ORs reflect changes in likelihood of choice of each criterion based on “best–worst” differences, relative to the reference criterion. In this instance, the reference criterion was relative safety. Alongside β or ORs, the model incorporates a standard deviation capturing unexplained variation around the mean (16). Based on this method, the thirteen criteria were ranked according to the preferences of Iran’s health system experts. Excel 2016 and Stata 17 software were employed for analyses in this section.

### Completing the IR-HIBOM elements

#### Determining the weight of relative importance of criteria

Coefficients obtained from the mixed logit analysis in the previous phase, scaled from 0 to 100, were used to determine the relative weights of the thirteen allocation criteria.

#### Determining and defining the framework of scoring levels of criteria and decision rules

Defining the scoring levels of the criteria and framework of scoring involved reviewing scoring levels of similar criteria in past models and studies. The study team, in several expert panels, finalized the scoring levels and framework of the model.

The process included:

1. Drafting scoring levels based on objectives, criterion nature, and available evidence.
2. Five iterations of review and editing by the expert panels (each comprising five specialists from various fields: health economics, health policy, health management, HTA, and epidemiology).
3. Finalizing scoring levels and framework.

In the first four sessions, three to four criteria were reviewed per session, and the scoring system and its framework were defined. The fifth session was dedicated to summarizing and finalizing all of the discussed items.

For new criteria (those covering social aspects), scoring levels and definitions were determined by the study team, aiming for precision and quantifiability. Three to five levels were assigned to each criterion based on its nature, and scores were allocated to each level. Due to the differing numbers of defined levels of criteria, scoring across the levels were normalized to the range of 0–1.

Some criteria used a straightforward approach (e.g., low, medium, high), while others included decision rules for flexibility and optimization.

### Model appraisal

Case examples of thirteen health technologies previously assessed (in 2021 and 2022) for insurance coverage by the HTA office of the Ministry of Health of Iran were used to appraise the potential utility of IR-HIBOM (17). These case examples were selected to represent a diversity of health care (e.g., medicines, supportive care, screening) and to probe the range of allocation criteria. The health technologies and their target groups and indications included:

1. HT 1: Medicine in the treatment of metastatic breast cancer
2. HT 2: Medicine in the treatment of metastatic breast cancer
3. HT 3: Ablation technology in the treatment of atrial fibrillation
4. HT 4: Ablation technology in the treatment of atrial fibrillation
5. HT 5: Rehabilitation in the management of relapsing remitting multiple sclerosis (RRMS)
6. HT 6: Medicine in the treatment of advanced melanoma
7. HT 7: Medicine in the treatment of advanced melanoma
8. HT 8: Medicine in the treatment of primary progressive multiple sclerosis (PPMS)
9. HT 9: Medicine in the treatment of relapsing remitting multiple sclerosis (RRMS)
10. HT 10: Technology for sedation in intensive care unit (ICU)
11. HT 11: Technology for sedation in intensive care unit (ICU)
12. HT 12: Technology in osteoporosis screening
13. HT 13: Technology in osteoporosis screening

Due to proprietary considerations, the generic and commercial names of these technologies are not identified. Model evaluation using the MCDA model was conducted through the simple additive weighting (SAW) technique. This method involves forming a decision matrix and calculating the final rank after normalizing and weighting the criteria (18).

The process included:

- Extracting and summarizing relevant general evidence from existing HTA reports.
- Determining the score of each technology in each criterion based on evidence and expert opinions of the study team.
- Completing the performance matrix of the model by incorporating these scores.
- Assigning a total weighted score to each technology based on the estimated importance weight of each criterion.
- Ranking all technologies was conducted based on their total weighted score. In this manner, technologies with higher total weighted scores were prioritized for resource allocation, while those with lower scores were assigned lower priorities.

To compare the rankings based on the IR-HIBOM method with those determined by cost-effectiveness criteria, we also ranked selected technologies based solely on their cost-effectiveness index (cost-effectiveness ratio). Given that the HTA reports for these technologies span different years, we calculated each technology’s average cost-effectiveness ratio to gross domestic product per capita ratio (i.e., ACER/GDP ratio) for the respective years of study. ACER values were calculated based on information present in the HTA reports.

## Results

### Final criteria

From the pool of potential allocation criteria, thirteen encompassing diverse aspects were ultimately selected in the initial phase. Table 1 shows a comprehensive list of the criteria along with their descriptions.

**Table 1.** Criteria and descriptions

	Criterion title	Description
1	Relative safety (relative rate of side effects of health technology)	Relative rate of side effects caused by the use of therapeutic health technology (here, we mean health technologies that are all confirmed safe and only the difference in the probability of their side effects is considered)
2	Efficacy	The effect of health technology on improving the disease state, life expectancy, and improving the patient’s quality of life
3	Cost-effectiveness	The cost of applying a health technology per unit of effectiveness (e.g., the cost per year of life years gained/QALY, …)
4	Budget impact	The budget required to provide the health technology according to the cost of the health technology and the patient population
5	Population size	The number of patients who need specific health technology to treat the disease
6	Access to alternative health technologies	The number of medicines or alternative health technologies available to treat a given disease
7	Severity of the disease	The fatality rate and level of disability of the disease
8	Age (pediatric/adults)	The age range of patients using the health technology (children or adults)
9	Work absenteeism	The impact of the disease on the absenteeism of working patients
10	Economic status of patients (poor/rich)	Example: higher prevalence of the desired disease among the poorer groups of the society
11	Daily care needs	Does the patient need daily care by family members or nurses?
12	Ease-of-use (acceptance by the patient)	Better acceptance of treatment by patients due to ease of receiving
13	Quality of available evidence	The quality of available scientific evidence regarding various aspects of the use of health technology, including safety and effectiveness

These criteria served as the foundation for subsequent phases in the study, providing a comprehensive framework for the development and evaluation of the IR-HIBOM.

### Determination and estimation of criteria importance weights

To ascertain and estimate the relative weights of the thirteen criteria, the preferences of health system experts were assessed using the BWS method. The resulting rankings and valuations of the criteria were derived from this process.

#### Characteristics of health system experts

The average age of the 163 health system experts was 59.5 years, ranging from 30 to 75 years. Men comprised 57.7 percent of the experts; health economists accounted for 18.4 percent, constituting the single largest share of the experts. Regarding professional work experience, 21.5 percent reported less than 5 years, while 20.3 percent had more than 25 years. Supplementary Table S1 provides a comprehensive breakdown of this group.

#### BWS findings and estimating the criteria’s importance weights

Table 2 presents the findings from the mixed logit analysis, including the frequency of each criterion being selected as the most and least important. ORs were computed for each criterion. The ORs were estimated in comparison to the first criterion in the dataset (i.e., relative safety), and the reported significance was based on this referent.

**Table 2.** BWS analysis results and ranking of study criteria

Criteria	Frequency of selection as best	Frequency of selection as worst	Odds ratio^1^	Standard error	Weight (adjusted based on 100)	Rank
Cost-effectiveness	432	32	5.58	0.45	30.51	1
Efficacy	344	26	3.58	0.27	19.58	2
Population size	268	85	1.87	0.14	10.24	3
Disease severity	198	68	1.51	0.11	8.24	4
Quality of evidence	171	94	1.19	0.08	6.52	5
Access to alternative health technologies	182	136	1.02	0.07	5.59	6
Relative safety	110	105	0.91	0.06	4.97	7
Budget impact	167	166	0.83	0.06	4.54	8
Economic status of patients	127	185	0.65	0.05	3.57	9
Age	38	244	0.38	0.03	2.09	10
Daily care needs	30	292	0.3	0.02	1.64	11
Work absenteeism	24	306	0.27	0.02	1.49	12
Ease-of-use (acceptance by the patient)	28	380	0.19	0.01	1.03	13

*Note:* Odds ratios reflect changes in likelihood of choice based on “best–worst” differences, relative to the reference criterion.

The criteria rankings place cost-effectiveness (OR = 5.58) as the most important criterion and ease-of-use (OR = 0.19) as the least important.

For the BWS results, the importance weight of each criterion was calculated based on the OR coefficients. The estimated importance weights, rescaled within the range of 0–100, are presented in Table 2, where cost-effectiveness holds the highest weight at 30.5 percent and ease-of-use has the lowest weight at 1 percent.

### Levels of criteria and framework of scoring

Following the determination of criterion weights, scoring levels were established, considering three to five levels assigned to each criterion. Adjustments were made within the range of 0–1 to normalize the scores. The detailed scoring levels for criteria are presented in Table 3, and additional explanations, including the scoring framework and the decision rules for two criteria, can be found in Table 4. The tables indicate that certain criteria in the developed model necessitate scoring by a specialized committee of the study team due to the diverse nature of health technologies.

**Table 3.** Scoring levels framework for criteria in the IR-HIBOM

Criteria and levels	Score	Adjusted score
*Efficacy*		
High efficacy compared to alternative health technologies	3	1
Moderate efficacy compared to alternative health technologies	2	0.67
Low efficacy compared to alternative health technologies	1	0.33
*Relative safety (incidence of side effects)*		
It has no side effects	6	1
Mild side effect with low incidence	5	0.83
Mild side effect with moderate incidence/moderate side effect with low incidence	4	0.67
Mild side effect with high incidence/moderate side effect with moderate incidence	3	0.5
Severe side effect with low incidence	2	0.33
Severe side effect with moderate incidence	1	0.17
*Population size (disease prevalence)*		
More than 500,000 patients	5	1
Between 100,000 and 500,000 patients	4	0.8
Between 50,000 and 100,000 patients	3	0.6
Between 10,000 and 50,000 patients	2	0.4
Less than 10,000 patients	1	0.2
*Quality of evidences*		
Systematic reviews with high quality of randomized controlled trials or health technology assessment with high quality therein systematic review has been performed (based on checklist of CASP and INAHTA)	5	1
Systematic reviews with medium or low quality of randomized controlled trials or heath technology assessment with medium or low quality therein systematic review has been performed (based on checklist of CASP and INAHTA)	4	0.8
Randomized controlled trials or clinical controlled trials with high quality or systematic review with high quality of other studies (based on CASP checklist)	3	0.6
Randomized controlled trials or clinical controlled trials with medium or low quality or systematic review with medium or low quality of other studies or cohort studies with any kind of quality (based on CASP checklist)	2	0.4
Other studies (cohort, case series, quasi-random studies, etc.) with any quality	1	0.2
*Access to alternative health technologies*		
Lack of access or weak access to alternative technologies for the target disease	3	1
Moderate (convenient) access to alternative technologies for the target disease	2	0.67
High access to alternative technologies for the target population	1	0.33
*Cost-effectiveness*		
Very good cost-effectiveness (cost per QALY less than 1 time GDP per capita)	4	1
Relatively good cost-effectiveness (cost per QALY between 1 and 2 times GDP per capita)	3	0.75
Low cost-effectiveness (cost per QALY between 2 and 3 times GDP per capita)	2	0.5
No cost-effectiveness (cost per QALY greater than 3 times GDP per capita)	1	0.25
*Budget impact*		
Significant savings	3	1
No significant change in additional costs or savings	2	0.67
Significant incremental costs	1	0.33
*Disease severity*		
High possibility of disease fatality	3	1
Moderate possibility of disease fatality	2	0.67
Low possibility of disease fatality	1	0.33
*Economic status of patients*		
The disease is prevalent in the lower income groups (higher and significant prevalence among the lower income groups)	3	1
The disease has the same prevalence in all income groups	2	0.67
The disease is prevalent in high-income groups (higher and significant prevalence among high-income groups)	1	0.33
*Age*		
Health technology is mainly used in children	3	1
Health technology is used in all age groups	2	0.67
Health technology is mainly used in the adults and elderly	1	0.33
*Daily care needs*		
The patient is fully dependent (needs full-time nursing)	3	1
The patient is somewhat dependent (needs part-time nursing)	2	0.67
The patient is independent (does not need nursing)	1	0.33
*Work absenteeism*		
The patient is unable to work or productivity is greatly reduced	3	1
The patient will not be able to return to his job or the productivity will decrease relatively	2	0.67
The patient will be able to work despite the disease or productivity will be temporarily reduced	1	0.33
*Ease-of-use/acceptance by the patient*		
High (easiness to receive is more than alternative health technologies/more preference than alternative health technologies)	3	1
Moderate (the ease of receiving alternative health technologies is the same/patients’ preferences are not clear or patients do not have a specific preference)	2	0.67
Low (less convenience compared to alternative health technologies/less preference compared to alternative health technologies)	1	0.33

**Table 4.** Instructions and decision rules for criteria scoring in the IR-HIBOM

Criteria	Scoring instructions and decision rules
Efficacy	Efficacy at the clinical trial level is the scoring basis Scoring based on the importance of clinical effectiveness (degree of importance of efficacy) is done by the committee and using evidence Efficacy in different groups of health technologies (therapeutic, diagnostic, preventive) is determined based on the characteristics of group Intermediate outcomes in therapeutic health technologies In diagnostic health technologies, sensitivity and specificity Chances of success in preventive health technologies
Relative safety	In this regard, it does not mean whether the health technologies are safe or not According to the type and degree of importance of the adverse effect, scoring is considered by the committee
Population size	When the range of the population size of the compared health technologies is in one group or two consecutive groups, in these cases, the classification should be rescaled based on the upper limit and lower limit of the interval.
Cost-effectiveness	If evidence of health technologies’ cost-effectiveness is available based on the ICER index compared to their best available comparator, ICER values are prioritized over ACER values in scoring due to their greater relevance in this context. If the prioritization involves two or more alternative health technologies, scoring will necessarily be based on their ICER values. In cases where the outcomes of the compared technologies are calculated based on the DALY averted unit, scoring will be similar to QALY. When the consequences of the evaluated technologies cannot be estimated using the QALY or DALY averted index and are instead derived based on natural units, scoring the technologies based on the cost per outcome unit occurs. This scoring considers the committee’s opinion regarding the status of alternative technologies and the value of each outcome unit produced as a result of the technology. ***Decision rules:*** When there is no information about the cost-effectiveness of the technology or there is insufficient information, the technology score for this criterion is considered 0 or 1 based on the opinion of the committee. When the health technology is not cost-effective at all, with the cost per QALY exceeding three times the GDP per capita and receiving a score of zero, the decision regarding resource allocation is made by the committee after evaluation, scoring, and final ranking. This decision takes into account the status of the health technology based on other criteria, such as the severity of the disease and access to alternative health technologies.
Budget impact	Scoring and considering the threshold based on: Total annual direct costs per patient Total annual drug cost per patient Total annual cost of disease-related health technologies per patient ***Decision rules:*** When the technology receives a score of 1 for its budget impact, indicating it incurs more than 100% additional costs compared to the alternative technology, it is excluded from the comparative evaluation of the model. Subsequently, the committee decides on the priority of allocating resources to this technology based on its state concerning other criteria, such as the severity of the disease and access to alternative health technologies.
Disease severity	High possibility of disease fatality: the possibility of death of patients in less than 1 year is higher than 90% High possibility of disease fatality: the possibility of death of the patient in 1 year is more than twice the normal death rate of based on age and sex High possibility of disease fatality: the possibility of death of the patient in 1 year is according to the normal death rate based on age and sex
Age	Scoring based on the average age or the most common age range in the country
Daily care needs	This criterion is leveled and scored based on the degree of dependence of people and patients in order to do their daily activities (activity daily living) For the leveling and scoring of technologies, general indicators can be used to check functional independence, such as the Barthel index, and in some cases, specific disease indicators can be used for this purpose.

Additional details of the scoring framework for each criterion are provided in the online supplementary file 2.

### Completing the performance matrix and appraising the model

The thirteen technologies were scored, drawing upon available evidence and applying the scoring levels of the thirteen criteria in the model accordingly.

The resulting performance matrix of the model is presented in Supplementary Table S2. Following the completion of the performance matrix, each technology’s total weighted score was computed within the model. Subsequently, a ranking was established, prioritizing thirteen health technologies with higher total scores for resource allocation. The scoring and rankings are detailed in Table 5.

**Table 5.** Appraisal of the IR-HIBOM and ranking of selected health technologies

Health technology	Scoring based on ACER/GDP per capita	Rank	Scoring based on IR-HIBOM	Rank
HT 12	The evidence is only identified in the form of costs for each true positive and true negative cases	–	82.39	1
HT 9	0.79	3	77.47	2
HT 13	The evidence is only identified in the form of costs for each true positive and True negative cases	–	76.2	3
HT 2	0.89	4	74.04	4
HT 3	0.23	2	73.31	5
HT 4	0.2	1	68.92	6
HT 8	1.45	6	68.12	7
HT 7	1.10	5	62.14	8
HT 11	Lack of sufficient evidence	–	49.55	9
HT 5	1.95	7	48.72	10
HT 6	11.63	9	48.67	11
HT 1	8.12	8	44.07	12
HT 10	Lack of sufficient evidence	–	42.41	13

Among the ranked technologies, HT 12 for osteoporosis screening secured the top priority with a score of 82.4. HT 10 for sedation in the ICU had the lowest priority with a score of 42.4.

To provide a comparative perspective on the evaluation results derived from the designed model and those based on cost-effectiveness criteria, Table 5 also displays the scores and rankings of technologies based only on the ACER/GDP per-capita criteria. This comparison reveals significant variation in the rankings between the two approaches. It is important to acknowledge that the evidence regarding the cost-effectiveness of the screening technologies HT 12 and HT 13 was presented based on the cost per true positive/negative cases identified, rendering practical comparability with other technologies in this aspect challenging.

In Iran, the primary authority responsible for the regulation, development, and revision of social health insurance packages is the Secretariat of the Supreme Council of Health Insurance. The results of this study have been presented to the Secretariat for its consideration for potential use in some form for helping to support future revisions of health insurance benefit packages.

## Discussion

In this study, a MCDA model, IR-HIBOM, was developed using a systematic approach, combining quantitative and qualitative methods across successive stages. The goal was to demonstrate a methodology for prioritizing health technologies for the allocation of healthcare financial resources and the formulation of health insurance benefit packages. The comprehensive nature of the model’s design involved determining relevant criteria, gauging perspectives of health system experts, assigning values, estimating the criteria’s relative importance weights, and establishing scoring levels and decision rules.

Ultimately, thirteen criteria were incorporated into the final modeling phase, encompassing various dimensions related to the model’s purpose. Various dimensions related to health technology nature, disease severity, economic characteristics, opportunity cost, equity considerations, and the quality of supporting evidence. In comparison to models with similar purposes, IR-HIBOM is relatively comprehensive in its scope of nonredundant criteria and diverse dimensions. Among other similar models, the Netherlands health technology prioritization model considered criteria such as disease burden, potential health benefits, patient numbers, health technology costs, long-term financial consequences, and impact on health system policies (19). Canada’s Evidence and Value: Impact on Decision Making (EVIDEM) HTA prioritization model uses criteria similar to those in the present model, that is, improving effectiveness, safety, alternative health technologies, budget impacts, completeness and consistency of available evidence, population size, disease severity, and cost-effectiveness (20). Lithuania’s prioritization model considered such criteria as health benefits, quality and quantity of evidence, evaluation timing, expected benefits for policymakers, and social, legal, and ethical considerations (21). Notably, IR-HIBOM combines quantitative and qualitative elements. A model developed by Mobinizadeh et al. in Iran in 2016 (11) and updated in 2021 (22) for selecting and prioritizing HTA topics shares notable similarities with the present model. Common criteria include health benefits at the population level, the size of the vulnerable population, availability of alternative technologies, budget impacts, financial protection, and the quality of evidence. “The Mobinizadeh et al. model incorporates safety criteria as a veto criterion (a condition or threshold in decision-making models, that can outright reject an alternative regardless of its performance in other criteria) and decision rules pertaining to scoring ‘uncertainty of cost-effectiveness’. The current model aligns closely with the Mobinizadeh et al. model, particularly regarding types of criteria and inclusion of decision rules.”

A distinctive feature of this study is the use of the BWS statistical method for weighting the allocation criteria. This method, involving a larger pool of experts specializing in health management and policymaking, sets it apart from similar studies. The model’s results reveal that the cost-effectiveness criterion holds the greatest weight, while the ease-of-use/acceptance of the health technology criterion is deemed least important. Due to variations in criteria and methodological differences in measuring relative weight, direct comparisons with other models, such as those of Mobinizadeh et al. and EVIDEM, present challenges. Notably, the analytic hierarchy process method used in these models differs fundamentally from the approach employed in this study.

Furthermore, this study contributes to the field by establishing and defining scoring levels within each criterion, considering their relative effects and minimizing overlap. The scoring scale varies across criteria, encompassing three, four, or five levels for each criterion, as appropriate, distinguishing it from Mobinizadeh et al. model, which employs a one to five scale, and the EVIDEM model, which adopts a zero to three scale for scoring or valuing each criterion (20;22).

This study incorporates decision rules for certain criteria to enhance the model’s capability and flexibility. These rules are particularly relevant to the cost-effectiveness and budget impact criteria, providing guidance for decision making under specific conditions. By considering factors such as the severity of the disease, access to alternative health technologies, and other prioritized criteria, decision rules ensure that a technology’s relative priority is not determined solely by traditional parameters used in determining cost-effectiveness or budget impact. Decision rules can vary in their application across different criteria, and there is potential for their consideration on a broader scale within the entire model. Models from England (23) the Netherlands (24), and Mobinizadeh et al. (22) also incorporate decision rules, particularly addressing the uncertainty of cost-effectiveness in their model. This approach aligns with a collaborative international effort to use decision rules in MCDA to support HTA (25).

For the evaluation of the final model, a diverse set of thirteen technologies was carefully selected to probe its utility, including of the allocation criteria and their levels, scoring framework, and decision rules. This approach facilitates a thorough assessment of the model’s effectiveness. Additionally, the results of prioritization utilizing the IR-HIBOM were compared to those from traditional approach based on cost-effectiveness, which revealed significant differences in rankings. Considering additional criteria explicitly through a prioritization model as demonstrated here can make a significant impact on prioritizing health technologies for resource allocation.

### Strengths and limitations

While efforts were made to address limitations prevalent in similar models, inherent challenges persist in MCDA models. Potentially overlapping criteria were carefully distinguished through precise definitions, scoring levels for each criterion, and scoring framework. However, some limitations, such as the inability to assess the quality of published HTA reports, remain.

A potential limitation of the IR-HIBOM model lies in its practical application and acceptability within routine decision-making processes. Due to its comprehensive nature, there may be concerns regarding the willingness of HTA bodies to adopt it as a standard approach. As noted earlier, the results of this study were presented to the Secretariat of the Supreme Council of Health Insurance, the primary authority responsible for regulating, developing, and revising social health insurance packages in Iran. Consideration and potential use of some form of this model by the Secretariat could support more objective, transparent, and routine revisions of health insurance benefit packages. However, further research is required to assess whether the benefits of using such a comprehensive model outweigh the potential challenges of implementing it in practical, real-world settings.

Despite these challenges, the study demonstrates certain strengths. Among these are the design of a MCDA model based on specialized concepts in health economics, along with the rationale for and integration of statistical and mathematical techniques. The use of innovative methods, such as the BWS method, enabled the participation of a larger, more diverse set of experts in the weighting process. Moreover, the inclusion of specialized expert panels in different stages of the research enhances the model’s credibility and applicability for HTA.

## Conclusion

The constraints of public budgets and the imperative to prioritize resource allocation for health technologies call for robust, transparent methodologies. Recognizing the need to account for a diverse, nonredundant set of criteria to inform resource allocation, employing a suitable framework to incorporate them and generate informed outputs becomes invaluable for decision making. Incorporating the preferences of different stakeholders can significantly influence related public policies. This study demonstrates the applicability of IR-HIBOM for health resource allocation within Iran, particularly for addressing the practical challenges of compiling and revising health benefit packages for a national health system.

These models should be viewed as decision-support tools. They can help to guide, though not replace, deliberative processes, serving as dynamic frameworks that can be updated over time to adapt to evolving constraints and opportunities within the health system. Ultimately, the application of this model will depend on the evolving decisions and policies of the relevant authorities, recognizing the need for further evaluation of its practical implementation in real-world settings.

## Supporting information

Darvishi et al. supplementary material 1 Darvishi et al. supplementary material

Darvishi et al. supplementary material 2 Darvishi et al. supplementary material

## Data Availability

The datasets generated and/or analyzed during this study are not publicly available but are available from the corresponding author on reasonable request.

## Supplementary material

The supplementary material for this article can be found at http://doi.org/10.1017/S0266462325100263.

## References

[1] Abelson J, Giacomini M, Lehoux P, Gauvin F-P. Bringing the public into health technology assessment and coverage policy decisions: From principles to practice. Health Policy. 2007;82(1):37–50. 16996637 10.1016/j.healthpol.2006.07.009

[2] Cleemput I, Neyt M, Thiry N, De Laet C, Leys M. Using threshold values for cost per quality-adjusted life-year gained in healthcare decisions. Int J Technol Assess Health Care 2011;27(1):71–76. 21262069 10.1017/S0266462310001194

[3] Musgrove P. Public spending on health care: How are different criteria related? Health Policy 1999;47(3):207–223. 10538919 10.1016/s0168-8510(99)00024-x

[4] Ubel PA. Pricing life: Why it’s time for health care rationing MIT Press; 2000.

[5] Youngkong S, Kapiriri L, Baltussen R. Setting priorities for health interventions in developing countries: A review of empirical studies. Trop Med Int Health. 2009;14(8):930–939. 19563479 10.1111/j.1365-3156.2009.02311.x

[6] Baltussen R, Niessen L. Priority setting of health interventions: The need for multi-criteria decision analysis. Cost Eff Resour Alloc. 2006;4:1–9. 16923181 10.1186/1478-7547-4-14 PMC1560167

[7] Belton V, Stewart T. Multiple criteria decision analysis: An integrated approach Springer Science & Business Media; 2002.

[8] Doshmangir L, Bazyar M, Majdzadeh R, Takian A. So near, so far: Four decades of health policy reforms in Iran, achievements and challenges. Arch Iran Med. 2019;22(10):592–605. 31679362

[9] Arab-Zozani M, Sokhanvar M, Kakemam E, Didehban T, Hassanipour S. History of health technology assessment in Iran. Int J Technol Assess Health Care 2020;36(1):34–39. 31928553 10.1017/S0266462319003489

[10] Hassanzadeh A. Iran’s health policy and healthcare system: Achievements and challenges. In Social policy in Iran. London, England: Routledge; 1st Edition, 2021. p. 49–80.

[11] Mobinizadeh M, Raeissi P, Nasiripour AA, Olyaeemanesh A, Tabibi SJ. A model for priority setting of health technology assessment: The experience of AHP-TOPSIS combination approach. DARU J Pharm Sci. 2016;24:1–12. 10.1186/s40199-016-0148-7 PMC4827190 27068692

[12] Tromp N, Baltussen R. Mapping of multiple criteria for priority setting of health interventions: An aid for decision makers. BMC Health Serv Res. 2012;12:1–7. 23234463 10.1186/1472-6963-12-454 PMC3565954

[13] Darvishi A, Daroudi R, Yaseri M, Sari AA. Public preferences regarding the priority setting criteria of health interventions for budget allocation: Results of a survey of Iranian adults. BMC Public Health. 2022;22(1):2038. 36344950 10.1186/s12889-022-14404-1 PMC9640781

[14] Flynn TN. Valuing citizen and patient preferences in health: Recent developments in three types of best–worst scaling. Expert Rev Pharmacoecon Outcomes Res 2010;10(3):259–267. 20545591 10.1586/erp.10.29

[15] Louviere JJ, Flynn TN, Marley AAJ. Best-worst scaling: Theory, methods and applications. Cambridge, England: Cambridge University Press; 2015.

[16] Guo Q, Shen J. A comparison between mixed logit model and latent class logit model for multi-profile best-worst scaling: Evidence from mobile payment choice dataset. Appl Econ Lett. 2022;29(14):1300–1305.

[17] Iran HTA Ofiice of the Ministry of Health and Medical Education. Health technology assessment reports; 2019.

[18] Hwang C-L, Yoon K. Methods for Multiple Attribute Decision Making. In: Multiple Attribute Decision Making. Lecture Notes in Economics and Mathematical Systems, 1981 vol 186. Berlin, Heidelberg: Springer. 10.1007/978-3-642-48318-9_3

[19] Oortwijn W, Banta D, Vondeling H, Bouter L. Identification and priority setting for health technology assessment in the Netherlands: Actors and activities. Health Policy 1999;47(3):241–253. 10538921 10.1016/s0168-8510(99)00020-2

[20] Goetghebeur MM, Wagner M, Khoury H, Rindress D, Grégoire J-P, Deal C. Combining multi-criteria decision analysis, ethics and health technology assessment: Applying the EVIDEM decision making framework to growth hormone for turner syndrome patients. Cost Eff Resour Alloc. 2010;1:8–15. 10.1186/1478-7547-8-4 PMC2856527 20377888

[21] Jankauskiene D, Petronyte G. A model for HTA priority setting: Experience in Lithuania. Int J Technol Assess Health Care 2013;29(4):450–455. 24290339 10.1017/S0266462313000470

[22] Mobinizadeh M, Mohamadi E, Arman H, Nasiripour A, Olyaeemanesh A, Mohamadi S. Topic selection for health technology assessment: An approach combining multiple attribute decision making and decision rules. Med J Islamic Repub Iran. 2021;35:40. 10.47176/mjiri.35.40 PMC8236113 34211942

[23] National Institute for Health and Care Excellence. NICE process and methods guides. Guide to the methods of technology appraisal 2013. London: National Institute for Health and Care Excellence (NICE); 2013.

[24] Nederland Z. Kosteneffectiviteit in de praktijk (cost-effectiveness analysis in practice). Diemen, The Netherlands: Zorginstituut Nederland; 2015.

[25] Baltussen R, Marsh K, Thokala P, et al. Multi-criteria decision analysis to support health technology assessment agencies: Benefits, limitations, and the way forward. Value in Health. 2019;22(11):1283–1288. 31708065 10.1016/j.jval.2019.06.014

