# Supplementary material for: Developing the Iranian health insurance benefit optimization model – the IR-HIBOM: a multicriteria decision analysis with decision rules for designing basic health insurance benefit packages
Source: Int J Technol Assess Health Care. 2025 Jul 21;41(1):e50. doi: 10.1017/S0266462325100263 (PMC12322855; doi:10.1017/S0266462325100263)
Supplement: Darvishi et al. supplementary material 1 — Darvishi et al. supplementary material [file S0266462325100263sup001.docx]

**Supplementary file 1**

**Questionnaire for Preferences assessment of health management experts**

**Introduction**

**In the name of God**

In all countries, government and health insurance resources are limited, preventing comprehensive coverage of all medicines and medical health technologies. Consequently, there is a need to prioritize therapeutic health technologies, focusing on those of higher importance.

Different drugs and therapeutic health technologies exhibit distinct "characteristics." For instance, some drugs are more effective but come with higher costs. Medications vary in their application, targeting severe or mild diseases, as well as specific age groups such as the elderly or children. Additionally, the availability of alternatives differs among drugs. Given these variations, it becomes imperative to identify and assess the importance of each characteristic, enabling the prioritization of health technologies accordingly.

The present questionnaire was crafted to facilitate a study aimed at determining the significance of specific features for prioritizing drugs and therapeutic health technologies. This study seeks to understand, from the perspective of stakeholders and experts within Iran's health system, the criteria and their respective levels of importance governing the prioritization of health technologies for government financial coverage.

The questionnaire comprises two main parts:

**Part 1: BWS questions and Choice Tasks**

Question: Suppose the Iran health insurance has a specific amount of funds and intends to allocate them between two medicines, covering only one of them. Considering that the allocation of the available budget involves considering various characteristics of the medicines and the treated disease, assume that the two medicines are comparable in all aspects except for four distinct criteria.

Now, in your opinion, which of the following four criteria should be accorded the "highest importance," and which one should be assigned the "lowest importance" in the process of budget allocation between the two medicines?

**Choice Task 1:**

| **Criterion and definition** | **Best** | **Worst** |
| --- | --- | --- |
| **Relative safety (relative rate of side effects of health technologies)**  Definition: The relative amount of side effects caused by the use of a therapeutic health technology (here, we mean health technologies that are all approved for safety, and only the difference in the probability of their side effects is considered) | **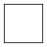** | **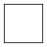** |
| **Cost-effectiveness**  Definition: The cost of applying a health technology per unit of effectiveness (for example, the cost per life year gained by the patient or QALY) | **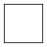** | **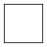** |
| **Daily care needs**  Definition: Does the patient under drug treatment need daily care by caregiver (family members or nurses) or not? | **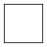** | **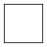** |
| **Ease of use (acceptance by the patient)**  Definition: The rate of acceptance of the health technology by the patient. Example: More acceptance of pen insulin by the patient compared to injection insulin | **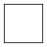** | **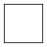** |

**Choice Task 2:**

| **Criterion and definition** | **Best** | **Worst** |
| --- | --- | --- |
| **Relative safety (relative rate of side effects of health technologies)**  Definition: The relative amount of side effects caused by the use of a therapeutic health technology (here, we mean health technologies that are all approved for safety, and only the difference in the probability of their side effects is considered) | **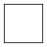** | **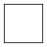** |
| **Access to alternative health technologies**  Definition: The frequency of drugs or alternative health technologies available to treat a given disease | **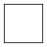** | **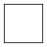** |
| **Work absenteeism**  Definition: The extent of the impact of the disease on the absenteeism of working patients | **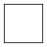** | **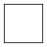** |
| **The quality of the available evidence**  Definition: The quality of the available scientific evidence regarding the various aspects of the use of intervention, including safety and effectiveness, etc. | **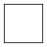** | **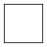** |

**Choice Task 3:**

| **Criterion and definition** | **Best** | **Worst** |
| --- | --- | --- |
| **Population size**  Definition: The number of patients who need specific health technology to treat the disease | **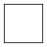** | **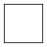** |
| **Disease severity**  Definition: Fatality rate and disability level of the disease | **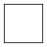** | **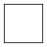** |
| **Work absenteeism**  Definition: The extent of the impact of the disease on the absenteeism of working patients | **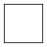** | **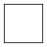** |
| **Ease of use (acceptance by the patient)**  Definition: The rate of acceptance of the health technology by the patient. Example: More acceptance of pen insulin by the patient compared to injection insulin | **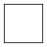** | **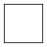** |

**Choice Task 4:**

| **Criterion and definition** | **Best** | **Worst** |
| --- | --- | --- |
| **Population size**  Definition: The number of patients who need specific health technology to treat the disease | **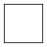** | **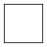** |
| **Economic status of patients (poor/rich)**  Definition: The prevalence of the desired disease is more among the poorer groups of the society or the richer groups of the society | **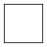** | **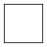** |
| **Daily care needs**  Definition: Does the patient under drug treatment need daily care by caregiver (family members or nurses) or not? | **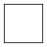** | **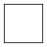** |
| **The quality of the available evidence**  Definition: The quality of the available scientific evidence regarding the various aspects of the use of intervention, including safety and effectiveness, etc. | **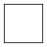** | **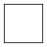** |

**Choice Task 5:**

| **Criterion and definition** | **Best** | **Worst** |
| --- | --- | --- |
| **Cost-effectiveness**  Definition: The cost of applying an health technology per unit of effectiveness (for example, the cost per life year gained by the patient or QALY) | **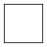** | **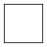** |
| **Age**  Definition: The age range of patients utilizing the health technology (e.g., children or adults) | **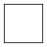** | **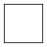** |
| **Work absenteeism**  Definition: The extent of the impact of the disease on the absenteeism of working patients | **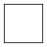** | **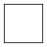** |
| **Economic status of patients (poor/rich)**  Definition: The prevalence of the desired disease is more among the poorer groups of the society or the richer groups of the society | **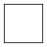** | **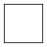** |

**Choice Task 6:**

| **Criterion and definition** | **Best** | **Worst** |
| --- | --- | --- |
| **Relative safety (relative rate of side effects of health technologies)**  Definition: The relative amount of side effects caused by the use of a therapeutic health technology (here, we mean health technologies that are all approved for safety, and only the difference in the probability of their side effects is considered) | **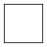** | **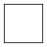** |
| **Efficacy**  Definition: The efficacy of therapeutic health technology on the improvement of the disease, longevity and quality of life of the patient | **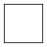** | **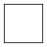** |
| **Disease severity**  Definition: Fatality rate and disability level of the disease | **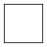** | **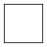** |
| **Economic status of patients (poor/rich)**  Definition: The prevalence of the desired disease is more among the poorer groups of the society or the richer groups of the society | **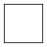** | **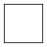** |

**Choice Task 7:**

| **Criterion and definition** | **Best** | **Worst** |
| --- | --- | --- |
| **Cost-effectiveness**  Definition: The cost of applying an health technology per unit of effectiveness (for example, the cost per life year gained by the patient or QALY) | **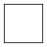** | **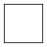** |
| **Budget Impacts**  Definition: The budget required to provide the health technology according to the cost of the health technology and the population of patients who use it | **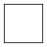** | **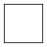** |
| **Disease severity**  Definition: Fatality rate and disability level of the disease | **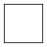** | **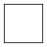** |
| **The quality of the available evidence**  Definition: The quality of the available scientific evidence regarding the various aspects of the use of intervention, including safety and effectiveness, etc. | **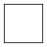** | **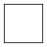** |

**Choice Task 8:**

| **Criterion and definition** | **Best** | **Worst** |
| --- | --- | --- |
| **Efficacy**  Definition: The efficacy of therapeutic health technology on the improvement of the disease, longevity and quality of life of the patient | **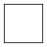** | **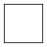** |
| **Age**  Definition: The age range of patients utilizing the health technology (e.g., children or adults) | **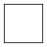** | **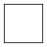** |
| **Ease of use (acceptance by the patient)**  Definition: The rate of acceptance of the health technology by the patient. Example: More acceptance of pen insulin by the patient compared to injection insulin | **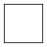** | **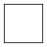** |
| **The quality of the available evidence**  Definition: The quality of the available scientific evidence regarding the various aspects of the use of intervention, including safety and effectiveness, etc. | **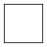** | **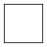** |

**Choice Task 9:**

| **Criterion and definition** | **Best** | **Worst** |
| --- | --- | --- |
| **Relative safety (relative rate of side effects of health technologies)**  Definition: The relative amount of side effects caused by the use of a therapeutic health technology (here, we mean health technologies that are all approved for safety, and only the difference in the probability of their side effects is considered) | **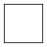** | **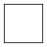** |
| **Budget Impacts**  Definition: The budget required to provide the health technology according to the cost of the health technology and the population of patients who use it | **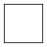** | **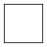** |
| **Population size**  Definition: The number of patients who need specific health technology to treat the disease | **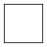** | **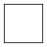** |
| **Age**  Definition: The age range of patients utilizing the health technology (e.g., children or adults) | **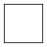** | **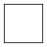** |

**Choice Task 10:**

| **Criterion and definition** | **Best** | **Worst** |
| --- | --- | --- |
| **Access to alternative health technologies**  Definition: The frequency of drugs or alternative health technologies available to treat a given disease | **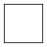** | **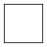** |
| **Disease severity**  Definition: Fatality rate and disability level of the disease | **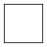** | **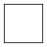** |
| **Age**  Definition: The age range of patients utilizing the health technology (e.g., children or adults) | **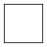** | **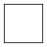** |
| **Daily care needs**  Definition: Does the patient under drug treatment need daily care by caregiver (family members or nurses) or not? | **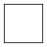** | **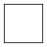** |

**Choice Task 11:**

| **Criterion and definition** | **Best** | **Worst** |
| --- | --- | --- |
| **Efficacy**  Definition: The efficacy of therapeutic health technology on the improvement of the disease, longevity and quality of life of the patient | **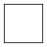** | **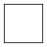** |
| **Cost-effectiveness**  Definition: The cost of applying an health technology per unit of effectiveness (for example, the cost per life year gained by the patient or QALY) | **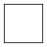** | **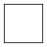** |
| **Population size**  Definition: The number of patients who need specific health technology to treat the disease | **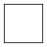** | **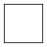** |
| **Access to alternative health technologies**  Definition: The frequency of drugs or alternative health technologies available to treat a given disease | **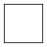** | **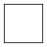** |

**Choice Task 12:**

| **Criterion and definition** | **Best** | **Worst** |
| --- | --- | --- |
| **Efficacy**  Definition: The efficacy of therapeutic health technology on the improvement of the disease, longevity and quality of life of the patient | **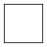** | **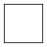** |
| **Budget Impacts**  Definition: The budget required to provide the health technology according to the cost of the health technology and the population of patients who use it | **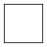** | **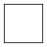** |
| **Work absenteeism**  Definition: The extent of the impact of the disease on the absenteeism of working patients | **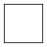** | **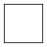** |
| **Daily care needs**  Definition: Does the patient under drug treatment need daily care by caregiver (family members or nurses) or not? | **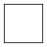** | **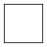** |

**Choice Task 13:**

| **Criterion and definition** | **Best** | **Worst** |
| --- | --- | --- |
| **Budget Impacts**  Definition: The budget required to provide the health technology according to the cost of the health technology and the population of patients who use it | **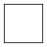** | **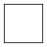** |
| **Access to alternative health technologies**  Definition: The frequency of drugs or alternative health technologies available to treat a given disease | **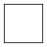** | **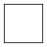** |
| **Economic status of patients (poor/rich)**  Definition: The prevalence of the desired disease is more among the poorer groups of the society or the richer groups of the society |  |  |
| **Ease of use (acceptance by the patient)**  Definition: The rate of acceptance of the health technology by the patient. Example: More acceptance of pen insulin by the patient compared to injection insulin |  |  |

**Table S1. Characteristics of participants in the BWS method survey**

| **Variable** | **Mean** | **SD** |
| --- | --- | --- |
| **Age** | 59.54 | 9.55 |
|  | **Freq** | **%** |
| **Sex (Male)** | 94 | 57.67 |
| **Expertise** | | |
| Health Economics | 30 | 18.4 |
| Health care Management | 26 | 15.95 |
| Health Policy | 19 | 11.66 |
| Pharmacoeconomics | 19 | 11.66 |
| Doctor of Medicine | 19 | 11.66 |
| Specialist doctor of medicine | 21 | 12.88 |
| Pharmacist | 19 | 11.66 |
| Others | 10 | 6.13 |
| **Work experience** | | |
| <5 years | 35 | 21.47 |
| 6-10 years | 36 | 22.09 |
| 11-15 years | 16 | 9.82 |
| 16-20 years | 24 | 14.72 |
| 21-25 years | 19 | 11.66 |
| >25 years | 33 | 20.25 |

**Table S2. Performance Matrix of the Model in the Appraising of 13 Selected Technologies**

| **Intervention/Technology** | **Cost-effectiveness** | **Efficacy** | **Population size** | **Disease severity** | **Quality of evidence** | **Access to alternative health technologies** | **Relative safety** | **Budget impact** | **Economic status of patients** | **Age** | **Daily care needs** | **Work absenteeism** | **Ease of use (acceptance by the patient)** |
| --- | --- | --- | --- | --- | --- | --- | --- | --- | --- | --- | --- | --- | --- |
| **HT 12** | 1 | 0.67 | 1 | 0.33 | 0.8 | 0.67 | 1 | 1 | 1 | 0.5 | 0.67 | 0.67 | 0.67 |
| **HT 9** | 1 | 0.67 | 0.6 | 0.67 | 1 | 0.67 | 0.5 | 1 | 0.33 | 1 | 0.33 | 0.33 | 0.67 |
| **HT 13** | 0.67 | 1 | 1 | 0.33 | 1 | 0.67 | 0.83 | 0.33 | 1 | 0.5 | 0.67 | 0.67 | 0.67 |
| **HT 2** | 1 | 0.33 | 0.2 | 1 | 1 | 0.67 | 0.67 | 1 | 0.67 | 1 | 1 | 1 | 1 |
| **HT 3** | 1 | 0.67 | 0.4 | 0.67 | 0.6 | 1 | 0.5 | 0.67 | 0.67 | 0.5 | 0.33 | 0.33 | 0.67 |
| **HT 4** | 1 | 0.33 | 0.4 | 0.67 | 0.8 | 1 | 0.67 | 0.67 | 0.67 | 0.5 | 0.33 | 0.33 | 0.67 |
| **HT 8** | 0.67 | 1 | 0.6 | 0.67 | 1 | 0.33 | 0.33 | 0.33 | 0.33 | 1 | 0.33 | 0.33 | 0.67 |
| **HT 7** | 0.67 | 0.33 | 0.2 | 1 | 1 | 1 | 0.33 | 1 | 0.67 | 0.5 | 0.67 | 1 | 0.67 |
| **HT 11** | 0.33 | 0.67 | 0.2 | 0.67 | 0.4 | 0.33 | 0.33 | 1 | 0.67 | 1 | 1 | 0.67 | 1 |
| **HT 5** | 0.33 | 0.33 | 0.6 | 0.67 | 0.4 | 1 | 1 | 0.33 | 0.33 | 1 | 0.67 | 0.67 | 0.33 |
| **HT 6** | 0 | 1 | 0.2 | 1 | 0.8 | 0.67 | 0.33 | 0.33 | 0.67 | 0.5 | 0.67 | 1 | 0.67 |
| **HT 1** | 0 | 0.67 | 0.2 | 1 | 0.8 | 0.67 | 0.33 | 0.33 | 0.67 | 1 | 1 | 1 | 1 |
| **HT 10** | 0 | 1 | 0.2 | 0.67 | 0.2 | 0.33 | 0.5 | 0.33 | 0.67 | 1 | 1 | 0.67 | 1 |
