# Supplementary material for: Developing the Iranian health insurance benefit optimization model – the IR-HIBOM: a multicriteria decision analysis with decision rules for designing basic health insurance benefit packages
Source: Int J Technol Assess Health Care. 2025 Jul 21;41(1):e50. doi: 10.1017/S0266462325100263 (PMC12322855; doi:10.1017/S0266462325100263)
Supplement: Darvishi et al. supplementary material 2 — Darvishi et al. supplementary material [file S0266462325100263sup002.docx]

**Supplementary file 2**

**Supplementary information of framework of scoring of criteria:**

The framework was meticulously compiled, taking into account criterion characteristics, definitions, and the extent of overlap with other criteria. It structures the determination of health technology scores based on the diversity of health technology and health technologies.

The criterion of *"access to alternative health technology"* requires judgments tailored to different disease groups, precluding a uniform approach.

*"Relative safety" criterion:* the model assesses health technology that are generally deemed safe. However, the scoring considers factors such as the type of side effect, the severity of occurrence, and the incidence rate to evaluate health technology in terms of relative safety.

*"Patient Population Size" Criterion:* The scoring levels are defined based on the prevalence of various diseases, ranging from less than 10,000 people to over 500,000 people. Considering that specific assessment of disease prevalence related to technologies may fall within certain defined levels, a more accurate scoring of the intervals between the prevalence levels of diseases is achievable through rescaling.

*"Quality and quantity of available scientific evidence" criterion:* Regarding various dimensions of health technology criterion, scoring levels were determined according to the evidence pyramid, accounting for the quality and quantity of evidence related to safety, effectiveness, and cost-effectiveness of the desired technology.

*"Cost-effectiveness" criterion:* Scoring levels have been standardized based on the cost per QALY (ICER or ACER). If ICER values are available for all health technologies being prioritized, based on comparisons with their best available comparators, the scoring of health technologies will be conducted using their ICER values. Otherwise, ACER values will be used to ensure comparability across technologies for different disease groups. Scoring levels are tied to cost-effectiveness thresholds derived from GDP per capita coefficients.

If the prioritization involves two or more alternative health technologies, scoring will necessarily be based on their ICER values.

Two decision rules have been included due to the importance of this criterion. According to the first rule, when there is insufficient or no information about the cost-effectiveness of the technology, the technology receives the lowest score (score 1), and the final decision is made by the committee based on the situation. The second rule comes into play when the health technology or technology is not cost-effective at all (for example: cost per QALY unit exceeds 3 times the GDP per capita) and receives a score of 1. In such cases, after evaluation, scoring, and final ranking, the decision regarding resource allocation is determined by the committee considering the health technology's status in terms of other criteria (e.g., disease severity, access to alternative health technology). These rules ensure that technologies with high scores in other criteria are not excluded from the ranking process based on limited evidence of cost-effectiveness or lack thereof, maintaining comparability. It's crucial to note the significance of the distance between ACER and 3 times the GDP per capita in the second rule.

*"Budget Impact" Criterion:* The scoring levels for the " Budget Impact" criterion is structured to assess the comparative nature of this criterion, encompassing saving money, incurring additional costs, and maintaining budget impacts compared to alternative technologies. A decision rule is outlined for the model, whereby if the desired technology scores 1 in terms of Budget Impact and incurs over 100 percent additional costs compared to the alternative technologies, it is excluded from the model's comparative evaluation. The committee then makes a decision regarding resource allocation priority for the technology, taking into account its status in other criteria (such as disease severity and access to alternative health technology).

*"Age " criterion:* Leveling and scoring are determined based on assigning higher scores to diseases affecting young ages and children, acknowledging the increased significance of diseases prevalent in reproductive age groups.

*"Disease severity" criterion:* Scoring levels are defined based on the lethality of the disease to avoid overlap with other criteria. The dimension of disability is considered in " Daily care needs" and "Work absenteeism " criteria, but not in the severity of the disease criterion. Scoring for disease severity is conducted based on degrees of normal age and gender mortality rates.

*"Work absenteeism" criterion:* Leveling and scoring are defined considering the reduction of productivity due to the disease, its duration (temporary or permanent), and its impact on the patient's ability to return to their previous job.

*"Ease of use (acceptance by the patient)"* criterion: Scoring levels are defined considering both dimensions: Ease of use or ease of application of technology and acceptance by the patient. This separation is due to the varying emphasis on either dimension depending on the technology. The scoring is determined according to the specific conditions associated with each dimension.
